# In this issue of the *Korean Journal of Women Health Nursing* March 2020

**DOI:** 10.4069/kjwhn.2020.03.26

**Published:** 2020-03-26

**Authors:** Sue Kim

**Affiliations:** College of Nursing, Yonsei University, Seoul, Korea

The March 2020 issue of the *Korean Journal of Women Health Nursing* includes articles relating to the following topic clusters:

· ***Data science***: An article on artificial intelligence (AI), machine learning, and deep learning, with their current use in nursing research, especially in Korea. Also, AI’s potential application for women’s health is presented.
· ***Reproductive health***: A study reports on mothers’ intention to vaccinate against the human papillomavirus (HPV) in an understudied subpopulation and elementary-aged boys. There are also two articles that focus on women with infertility, one on psychosocial factors affecting their quality of life, and the other on the role of uncertainty and spousal support for women receiving artificial reproductive technology.
· ***Maternal health***: A study on how pregnant women’s stress, depression, and social support affect maternal identity.
· ***Education in women’s health***: Simulation in nursing education is increasingly widely used. One article reports on a simulation-based postpartum hemorrhage education program’s effect on clinical performance, judgment, and learning satisfaction in nursing students. The other article presents how problem-based learning—integrative simulation practice was useful for critical thinking, problem-solving, and immersion.
· ***Professional roles***: An article on work performance and factors affecting job satisfaction in registered nurse-midwives.
· ***Women’s cancers***: A study on lifestyle, depression, and marital intimacy among breast cancer survivors.
· ***Gender analysis***: A secondary analysis study focusing on factors influencing gender differences in unmet health needs.

We hope you will enjoy the articles in this issue.

